# Additive Manufacturing of Ordered Polymer Nanostructures

**DOI:** 10.1002/adma.73395

**Published:** 2026-05-23

**Authors:** Di Wu, Kun Zhou, Kenny Lee, Yuan Xiu, Carol Hiu Wai Yan, Khoon S. Lim, Cyrille Boyer

**Affiliations:** ^1^ School of Chemical Engineering University of New South Wales Sydney New South Wales Australia; ^2^ Electron Microscope Unit Mark Wainwright Analytical Centre University of New South Wales Sydney New South Wales Australia; ^3^ School of Medical Sciences University of Sydney Sydney Australia

**Keywords:** 3D printing, block copolymers, nanostructures, photopolymerization, self‐assembly

## Abstract

Additive manufacturing (3D printing) allows the fabrication of complex 3D geometries, yet the integration of long‐range ordered nanostructures within printed materials remains a fundamental challenge. In vat photopolymerization, rapid crosslinking kinetics typically arrest block copolymers in kinetically trapped, disordered morphologies. Here, we introduce Polymerization‐Induced Arrangement of Nanostructures with Order‐tunability (PIANO), a strategy that overcomes this kinetic mismatch by decoupling nanoscale ordering from network formation. PIANO utilizes a mobility mediator, ethylene glycol, to enhance polymer chain mobility, enabling rapid in situ ordering, while maintaining a hydrogen‐bonding network capable of sustaining 3D printing stresses. This approach yields tunable lamellar and hexagonally packed cylindrical morphologies with domain spacings of 20–60 nm. Furthermore, ethylene glycol acts as a latent crosslinker during post‐printing annealing, locking the ordered nanostructure while enhancing macroscopic mechanical strength. By reconciling the divergent timescales of molecular self‐assembly and additive manufacturing, this strategy provides a robust platform for the hierarchical design of functional systems.

## Graphical abstract

A new 3D printing strategy, Polymerization‐Induced Arrangement of Nanostructures with Order‐tunability (PIANO), enables the formation of ordered nanostructures in polymer materials by enhancing chain mobility during photopolymerization, overcoming the kinetic arrest that leads to disordered morphologies in the conventional Polymerization‐Induced Microphase Separation (PIMS) 3D printing system.

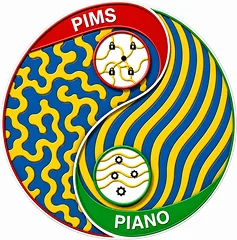

## 1 Introduction

Natural materials exhibit extraordinary properties through hierarchical organization, where nanoscale order is seamlessly integrated into macroscopic architectures. This multilevel sophistication is exemplified by the periodic brick‐and‐mortar structure of nacre, which imparts exceptional fracture toughness, the vivid structural coloration of butterfly wings, and the extraordinary toughness of spider silk [1]. Replicating this complexity in synthetic systems remains a central goal of advanced materials design [2, 3, 4, 5]. However, achieving such complexity is inherently challenging, as it requires precise control over structure formation across disparate length scales. Bridging nanoscale order with macroscopic form within a unified manufacturing framework, therefore represents a critical bottleneck in the realization of high‐performance, bio‐inspired materials.

While additive manufacturing (3D printing) has revolutionized macroscopic fabrication by offering unprecedented geometric freedom [6, 7, 8, 9, 10, 11, 12], achieving internal nanoscale order within printed objects remains largely elusive. Most 3D‐printed polymeric materials are isotropic and lack internal structural organization. Existing methods that achieve nanoscale features often rely on highly viscous, pre‐assembled block copolymer (BCP) inks, or specialized resins [13, 14, 15, 16, 17]. However, these systems are often constrained by low scalability, equipment dependence (e.g., direct ink writing (DIW) or two‐photon polymerization), and a reliance on preserving pre‐existing structures rather than enabling dynamic, printing‐induced formation. For instance, while we have previously demonstrated the preparation of ordered nanostructures using direct ink writing [16], achieving such order in high‐speed photopolymerization processes, such as stereolithography or liquid crystal display (LCD) printing, remains a significant challenge.

**FIGURE 1:**
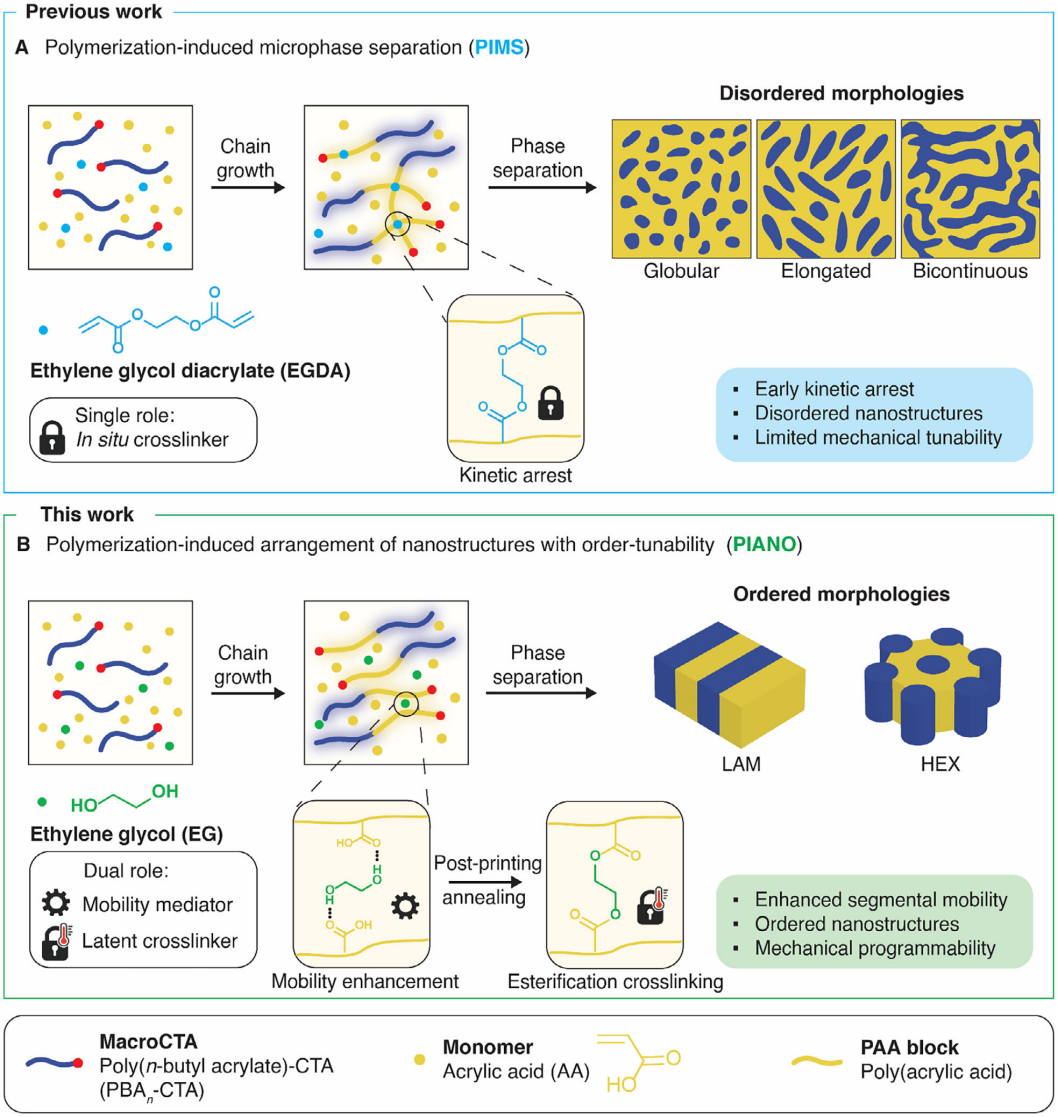
Schematic illustration of the competing kinetics and morphological outcomes in PIMS and PIANO during photopolymerization‐based 3D printing. (A) Conventional polymerization‐induced microphase separation (PIMS). In the presence of divinyl crosslinkers (ethylene glycol diacrylate, EGDA), the rapid formation of covalent networking kinetically arrests the developing BCP chains, which leads to “frozen” disordered morphologies that lack long‐range nanoscale order. (B) Polymerization‐induced arrangement of nanostructures with order‐tunability (PIANO). By replacing permanent crosslinkers with ethylene glycol (EG) (small‐molecule mobility mediator), PIANO decouples mobility regulation from crosslinked network formation, thereby opening a kinetic window that allows rapid evolution toward ordered lamellar (LAM), or hexagonally packed cylindrical (HEX) phases during the printing timeframe. Subsequent thermal annealing triggers esterification of the latent crosslinker (EG), locking the ordered nanostructure, and enhancing mechanical robustness.

Polymerization‐induced microphase separation (PIMS), introduced by Seo and Hillmyer, is an effective approach for generating nanoscale structures directly during the polymerization process [18, 19, 20, 21]. In a typical PIMS system, a macromolecular chain transfer agent (macroCTA) undergoes in situ chain extension in the presence of monomers and multi‐vinyl crosslinkers. As the reaction progresses, the resulting immiscible blocks trigger rapid phase separation, theoretically enabling control over nanoscale. Our group pioneered the implementation of PIMS within the 3D printing framework (Figure 1A) [22, 23, 24], successfully tuning nanodomain size and morphologies, and controlling macroscale structures [19, 25, 26, 27]. This versatility has facilitated the printing of functional materials, such as solid polymer electrolytes [28], ion‐exchange materials [29], and thermally insulating ceramics [30].

Despite their potential, 3D‐printed PIMS systems generally lack long‐range order due to the competing requirements of microphase separation and the rapid crosslinking. This ‘near‐instantaneous’ crosslinking typically arrests the morphology in a disordered, kinetically trapped state, such as bicontinuous, or globular structure [19, 31]. This limitation arises from a fundamental kinetic mismatch: while 3D photopolymerization requires completion within seconds at room temperature, the evolution of ordered copolymer morphologies necessitates sufficient segmental mobility over a comparable temporal window. In contrast, long‐range ordering in pre‐formed block copolymers typically demands hours of thermal or solvent annealing, rendering such approaches inherently incompatible with vat photopolymerization‐based 3D printing [31, 32, 33, 34, 35, 36].

To overcome this disparity, in this study, we introduce Polymerization‐Induced Arrangement of Nanostructures with Order‐tunability (PIANO), a strategy that enables the emergence of ordered nanostructures during photopolymerization by regulating chain mobility (Figure 1B). Distinct from conventional PIMS systems that rely on early network formation via multi‐vinyl crosslinkers, PIANO delays permanent network fixation while maintaining sufficient segmental mobility during chain growth through the incorporation of small‐molecule mobility mediators compatible with polymerizing phases (e.g., ethylene glycol). This decoupling extends the kinetic window for nanoscale ordering within the seconds‐long timescale of photopolymerization under ambient temperature. Using a commercial LCD 3D printer, we demonstrate the versatility of the PIANO approach through the direct 3D printing of ordered nanostructures, such as lamellar (LAM) or hexagonally packed cylinders (HEX), within printed materials. Thus, the PIANO strategy provides a versatile route for integrating ordered nanostructures into complex architectures via photopolymerization‐based 3D printing, opening new opportunities for advanced functional materials.

## 2 Results and Discussion

### 2.1 Resin Design

The PIANO resin comprises a macroCTA, a second monomer for block extension, a photoinitiator, and a small‐molecule mobility mediator (Figure 1B). In this design, poly(*n*‐butyl acrylate) macroCTAs (PBA*_n_*‐CTA), synthesized by reversible addition fragmentation chain transfer (RAFT) polymerization with controlled degrees of polymerization (*n* = 25, 50, or 100) were employed as the precursor for block copolymers (Figures S1–S3 and Table S1) [37]. Acrylic acid (AA) was selected as the second monomer due to its initial miscibility with the PBA block. Upon polymerization, the growing PAA blocks become strongly incompatible with the PBA precursor due to the positive Flory‐Huggins interaction parameter [23, 38], which provides the thermodynamic driving force for microphase separation. Central to the PIANO strategy, ethylene glycol (EG) was added in small quantities at a fixed [AA]/[EG] molar ratio of 6.4 (Table S2) and serves a dual role. Initially, it acts as a mobility mediator to facilitate rapid nanoscale ordering during photopolymerization while simultaneously forming a hydrogen‐bonding network with the poly(acrylic acid) (PAA) blocks; this network provides the structural integrity necessary to withstand printing‐induced stresses [39, 40, 41]. After printing, EG serves as a latent crosslinker. Thermal annealing activates esterification reactions between EG and PAA, resulting in permanent chemical crosslinking that locks in the nanostructure and enhances the mechanical properties. To ensure rapid radical generation compatible with open‐air LCD printing, diphenyl(2,4,6‐trimethylbenzoyl) phosphine oxide (TPO) was employed as the photoinitiator. Efficient polymerization and solidification of the PIANO resin were confirmed by the increase in vinyl bond conversion derived from Fourier transform infrared (FTIR) measurements (Figure S4) and the rapid modulus increase observed in photorheology experiments (Figure S5). These results collectively confirm quick monomer consumption and polymerization‐induced transition from a liquid to a solid state. PIANO formulations are denoted by the degree of polymerization (DP) of macroCTA and its weight fraction; for example, the 50/15 formulation contains 15.0 wt.% PBA_50_‐CTA, together with 74.0 wt.% AA monomer, 10.0 wt.% EG, and 1.0 wt.% TPO (Table S2).

### 2.2 Mechanistic Insights into the Emergence of Ordered Nanostructures by PIANO

To investigate how the PIANO strategy governs the formation of ordered nanostructures, we performed real‐time in situ small‐angle X‐ray scattering (SAXS) measurements. The resin was loaded into a transparent borosilicate capillary and irradiated with violet light (*λ*_max_ = 405 nm, *I*_0_ = 2 mW cm^−2^), replicating the photochemical conditions of 3D printing. Controlled irradiation intervals enabled real‐time monitoring of microphase separation kinetics and the structural transitions leading to highly ordered morphologies.

**FIGURE 2:**
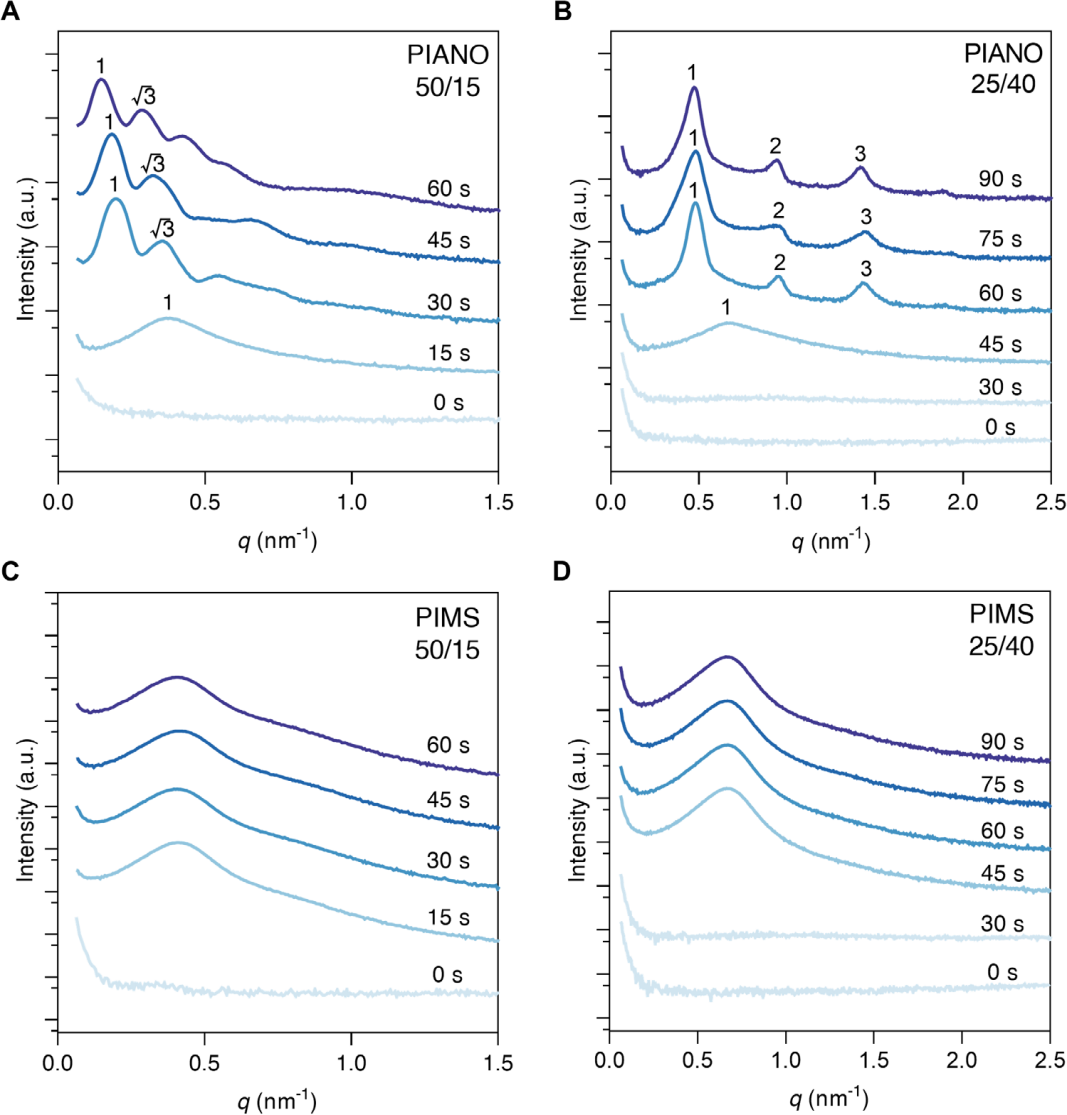
In situ small‐angle X‐ray scattering (SAXS) monitoring microphase separation in PIANO and PIMS systems. (A,B) Time‐resolved SAXS profiles of PIANO resins 50/15 and 25/40, showing the progressive emergence and intensification of multiple scattering peaks. The characteristic position ratios (*q*/q*) correspond to hexagonally packed cylindrical (HEX) and lamellar (LAM) morphologies, respectively, demonstrating rapid ordering during photopolymerization. (C,D) In contrast, PIMS systems 50/15 and 25/40 with equivalent macroCTA contents exhibit only a single, broad scattering peak through irradiation. This lack of long‐range order is attributed to early kinetic arrest induced by permanent network formation. *Note*: 50/15 denotes the resin containing 15.0 wt.% of PBA_50_‐CTA.

As shown in Figure 2A, the PIANO resin 50/15 was initially homogeneous, as evidenced by the absence of any scattering peaks at 0 s. Upon irradiation, a single broad scattering peak emerged at ∼0.38 nm^−1^, indicating the onset of nanoscale segregation. As exposure continued, a series of well‐defined Bragg reflections emerged by 30 s and gradually intensified. These peaks exhibited a specific *q*‐ratios of 1:√3, characteristic of a hexagonally packed cylinder (HEX) morphology [42, 43, 44, 45]. The primary scattering vector *q* ^*^ gradually shifted to lower *q* values with increasing irradiation time, signifying an increase in domain spacing as PAA blocks grew. The ordered structure stabilized after approximately 60 s, with a domain spacing of ∼42 nm (*d*_SAXS_ = 2π / *q* ^*^) and a scattering profile corresponding to HEX morphology [42, 43, 44, 45].

A similar progression was observed for the PIANO resin 25/40 in Figure 2B. After 45 s of irradiation, a single broad peak at *q* = 0.69 nm^−1^ indicated the formation of a disordered nanostructure. After 60 s, three distinct diffraction peaks appeared with ratios of 1:2:3, characteristic of a lamellar morphology [42, 43, 44]. In this case, the peak positions remained relatively stable as irradiation proceeded, confirming the domain spacing at ∼13 nm (*q* ^*^ = 0.48 nm^−1^).

For the 100/15 formulation in Figure S6, the ratio of secondary to primary SAXS peaks shifted from 2 toward √3 after 15 s irradiation. This suggests a rapid morphological evolution, where a lamellar intermediate state transitions into hexagonal cylinders as the degree of polymerization in the PAA block is increased during chain extension [46]. The calculated volume fractions of the PAA block in the 50/15 and 25/40 formulations were 0.79 and 0.50, respectively (Table S2). These results are consistent with the equilibrium morphologies typically observed in thermally annealed diblock copolymer systems, demonstrating that the PIANO strategy achieves equilibrium‐like order within seconds at room temperature [32, 38, 47].

To isolate the effect of the PIANO strategy, control PIMS systems were investigated using in situ SAXS by replacing EG with a divinyl crosslinker (ethylene glycol diacrylate, EGDA) at an equivalent molar ratio, which allows for a direct comparison between the PIANO approach and the traditional kinetically arrested PIMS approach (Table S3). In stark contrast to the ordered phases observed via PIANO, the corresponding PIMS systems 50/15 and 25/40 shown in Figure 2C,D exhibited only broad, singular scattering signals at a higher *q* value, with no detectable secondary peaks. Throughout the entire polymerization, these systems showed no secondary peaks or detectable peak shifts, a behavior that aligns with previous reports on cross‐linked PIMS materials [18, 19, 20, 23]. These observations confirm that without the mobility mediator, structural evolution is kinetically arrested, resulting in a lack of long‐range order. Conversely, the PIANO strategy enhances chain mobility, enabling the development of highly ordered arrangements within tens of seconds. Because this process occurs under mild, light‐mediated conditions, it is inherently compatible with high‐speed vat photopolymerization 3D printing. By bypassing the traditional requirements for long‐term solvent evaporation or high‐temperature thermal annealing [32, 33], this approach allows for spontaneous structural ordering during the layer‐by‐layer fabrication process. Furthermore, the ability to independently tune morphology (e.g., LAM vs. HEX) and domain spacing through formulation design demonstrates the potential of PIANO as a versatile platform for fabricating 3D‐printed materials with hierarchical structural control.

### 2.3 Application of PIANO Strategy in 3D Printing

**FIGURE 3:**
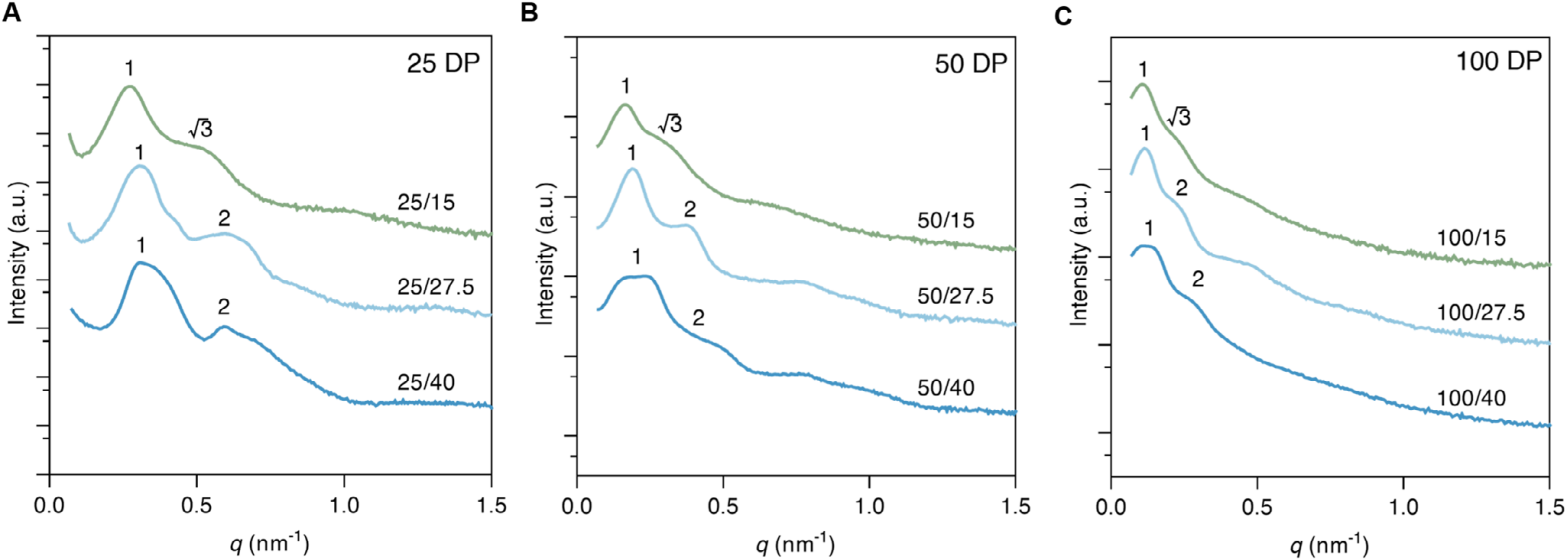
Small‐angle X‐ray scattering (SAXS) characterization of 3D printed PIANO film samples. The samples were prepared by resins containing different loadings (15.0, 27.5, and 40.0 wt.%) of (A) PBA_25_‐CTA, (B) PBA_50_‐CTA, or (C) PBA_100_‐CTA. *Note*: 50/15 denotes the resin containing 15.0 wt.% of PBA_50_‐CTA.

Building on these preliminary SAXS results, we applied the PIANO strategy to vat photopolymerization 3D printing. Using a commercial LCD printer (Anycubic Photon Mono SE, *λ*_max_ = 405 nm, *I*_0_ = 2 mW cm^−2^), we fabricated a series of simple objects to evaluate the printability of these PIANO resins (See SI for detailed procedures: “3D printing setup and procedure” section). Following the printing process, all samples underwent a 30‐min post‐curing treatment to ensure complete monomer conversion as determined by Fourier Transform Near‐Infrared (FTNIR) spectroscopy (Figure S7). Representative 3D‐printed objects were obtained with high geometric fidelity, demonstrating the good printability of the PIANO resins (Figure S8). To characterize the internal nanostructures of these materials, film samples (0.2 mm thickness) were printed and analyzed using SAXS. The resulting SAXS profiles for nine distinct PIANO formulations prepared by systematically varying both the DPs (25, 50, and 100) and loadings (15.0, 27.5, and 40.0 wt.%) of macroCTA are displayed in Figure 3.

As shown in Figure 3A, the 25/15 sample, prepared with 15.0 wt.% of PBA_25_‐CTA, exhibited a distinct primary peak at q^*^ ≈ 0.28 nm^−1^ and a broad shoulder at √3*q* ^*^. This *q*‐ratio of 1:√3 indicates the presence of an ordered HEX morphology within the 3D‐printed film [42, 43, 44], with a calculated domain spacing of 22 nm. As the macro‐CTA loading increased to 27.5 and 40.0 wt.%, the primary peak slightly shifted to q^*^ ≈ 0.31 nm^−^^1^ (20 nm) and the secondary peak appeared at 2q^*^, corresponding to a LAM morphology [42, 43, 44]. This morphological evolution from HEX to LAM directly correlates with the decreasing volume fraction of the PAA block (*f*_PAA_ = 0.79, 0.64, and 0.50 with increased macroCTA loadings for the three formulations; Table S2). These results are consistent with the established self‐assembly behavior of typical thermally annealed diblock copolymers [32, 38, 47], suggesting that the PIANO strategy allows the system to evolve toward thermodynamically favored states, despite the rapid kinetics of the 3D printing process.

As the macroCTA chain length increased from DP 25 to 50 (Figure 3B), the primary peaks shifted toward lower *q* values, indicating larger domain spacings. For instance, the 50/15 sample displayed a main peak at *q* ^*^ ≈ 0.17 nm^−^^1^, corresponding to an increased domain spacing of ∼ 37 nm, compared to 33 nm for 50/27.5 and 31 nm for 50/40. Notably, the 50/40 sample exhibited a broader primary peak than those with lower DP or lower macroCTA loading. This broadening can be attributed to the higher resin viscosity and lower chain mobility (Table S4), which can kinetically hinder long‐range structural evolution and decrease the grain size of the ordered domains. A similar trend was observed with the even longer PBA_100_‐CTA series (Figure 3C). The primary peaks shifted further toward lower *q* values, reflecting an increase in domain spacing ranging from 52 to 57 nm. Although the secondary reflections in this series appeared as broad shoulders, suggesting a decrease in long‐range order for the largest molecular weights, the patterns still clearly indicate the successful development of ordered nanostructures. Similar to 50/40, the broad scattering peak observed for 100/40 suggests that high macroCTA loading combined with increased chain length reduced the chain mobility, thereby limiting nanoscale ordering.

To further explore the morphological evolution accessible within the PIANO system, formulations with a reduced PBA volume fraction were investigated (Table S2). According to classical block copolymer phase behavior, decreasing the volume fraction of the minority block is expected to drive the morphology transition toward higher interfacial curvature, ultimately leading to spherical domains under near‐equilibrium conditions. However, as the PBA volume fraction decreases to 0.14 and 0.11, the SAXS profiles in Figure S9 exhibit multiple scattering peaks at *q* ^*^ and √3*q* ^*^, still indicative of hexagonally packed cylindrical domains. This behavior can be rationalized by the kinetic nature of the PIANO process. In contrast to equilibrium self‐assembly, nanostructure formation in PIANO occurs concurrently with photopolymerization, during which chain mobility rapidly decreases as conversion proceeds. As a result, the system becomes kinetically arrested prior to accessing the high interfacial curvature states required for spherical morphologies. These results highlight that the accessible morphologies in PIANO are governed by a balance between thermodynamic driving forces and kinetically limited chain mobility, rather than equilibrium phase behavior alone.

To more comprehensively delineate the kinetic window for nanoscale ordering in the PIANO process, formulation parameters were systematically varied to modulate chain mobility and polymerization kinetics. First, to isolate the role of EG in enhancing segmental mobility, the EG weight fraction was varied from 0 to 15 wt.% in the 100/15 formulation (Table S5). As shown in Figure S10A, increasing EG content leads to a progressive sharpening of the primary SAXS peak and the gradual emergence of higher‐order features, demonstrating a monotonic improvement in nanoscale ordering with enhanced segmental mobility. Second, beyond mobility modulation alone, comparable improvements in nanoscale ordering can be achieved by regulating the polymerization kinetics. In the 100/40 system, increasing EG content to enhance mobility or reducing the TPO concentration to slow chain growth resulted in sharper primary SAXS peaks and improved long‐range order (Figure S10B and Table S5). While increased EG content facilitates chain diffusion by lowering viscosity, reduced TPO concentration extends the temporal window available for structural organization by moderating the polymerization rate. However, these optimizations entail practical trade‐offs: excessive EG compromises the mechanical robustness and structural integrity, whereas reduced TPO concentration lowers the achievable printing speed.

Together, these results demonstrate that the formation of ordered nanostructures in the 3D‐printed PIANO system is a dynamic, kinetically regulated process. Morphological development is governed by a fundamental competition: while the thermodynamic driving force for separation increases with chain length, segmental mobility simultaneously decreases due to rising viscosity (Table S4). Achieving long‐range order thus requires kinetic matching, i.e., synchronizing the rate of chain growth with the rate of segmental motion. By jointly tuning internal formulation factors (EG content, macroCTA degree of polymerization) and external printing parameters (polymerization rate), the PIANO strategy enables rapid, cooperative ordering across a broad range of domain sizes (Table 1). Furthermore, consistent with this kinetic picture, the SAXS‐derived domain spacing is proportional to the total degree of polymerization across different macroCTA loadings following the power law *d* ∼ *N^δ^* [48], as revealed by log‐log analysis (Figure S11). The scaling exponent of ∼0.69 is close to the strong‐segregation limit from theory of microphase‐separated block copolymers [38], suggesting pronounced chain stretching consistent with a strong‐segregation‐like regime in this system. This pronounced dependence enables predictable and tunable control over the characteristic length scale of the ordered nanostructures through chain‐length design.

**TABLE 1:** Summary of SAXS and TEM results of 3D printed PIANO samples.

Nos.	Resins	MacroCTA	MacroCTA weight percentage (wt.%)	*d*_SAXS_ ^a^ (nm)	*d*_TEM_ ^b^ (nm)	Morphology
1	25/15	PBA_25_‐CTA	15.0	22	24	HEX
2	25/27.5		27.5	20	23	LAM
3	25/40		40.0	20	22	LAM
4	50/15	PBA_50_‐CTA	15.0	37	38	HEX
5	50/27.5		27.5	33	37	LAM
6	50/40		40.0	31	32	LAM
7	100/15	PBA_100_‐CTA	15.0	57	77	HEX
8	100/27.5		27.5	52	58	LAM
9	100/40		40.0	52	57	LAM

^a^ *d*_SAXS_ values were obtained by 2π/*q* ^*^ for both LAM and HEX morphologies (details found in SAXS section and Figure S12).

^b^ *d*_TEM_ values were measured by TEM images. For HEX nanostructures, *d*_TEM_ was obtained by converting the measured center‐to‐center distance between adjacent cylinders (*d*_c–c_) into the distance from a cylinder center to the nearest lattice plane (*d*_c–p_), using geometrical relationships (Figure S12).

To visualize the internal ordered nanostructures, transmission electron microscopy (TEM) was performed on ultramicrotomed cross‐sections (≈ 70–100 nm) of 3D printed PIANO cubes (2 mm × 2 mm × 2 mm). To ensure sufficient rigidity for the ultramicrotome, the samples were thermally treated at 120°C for 16 h. This annealing step did not alter the nanoscale morphology, as confirmed by SAXS profiles which showed identical peak positions and intensities before and after treatment (Figure S13).

**FIGURE 4:**
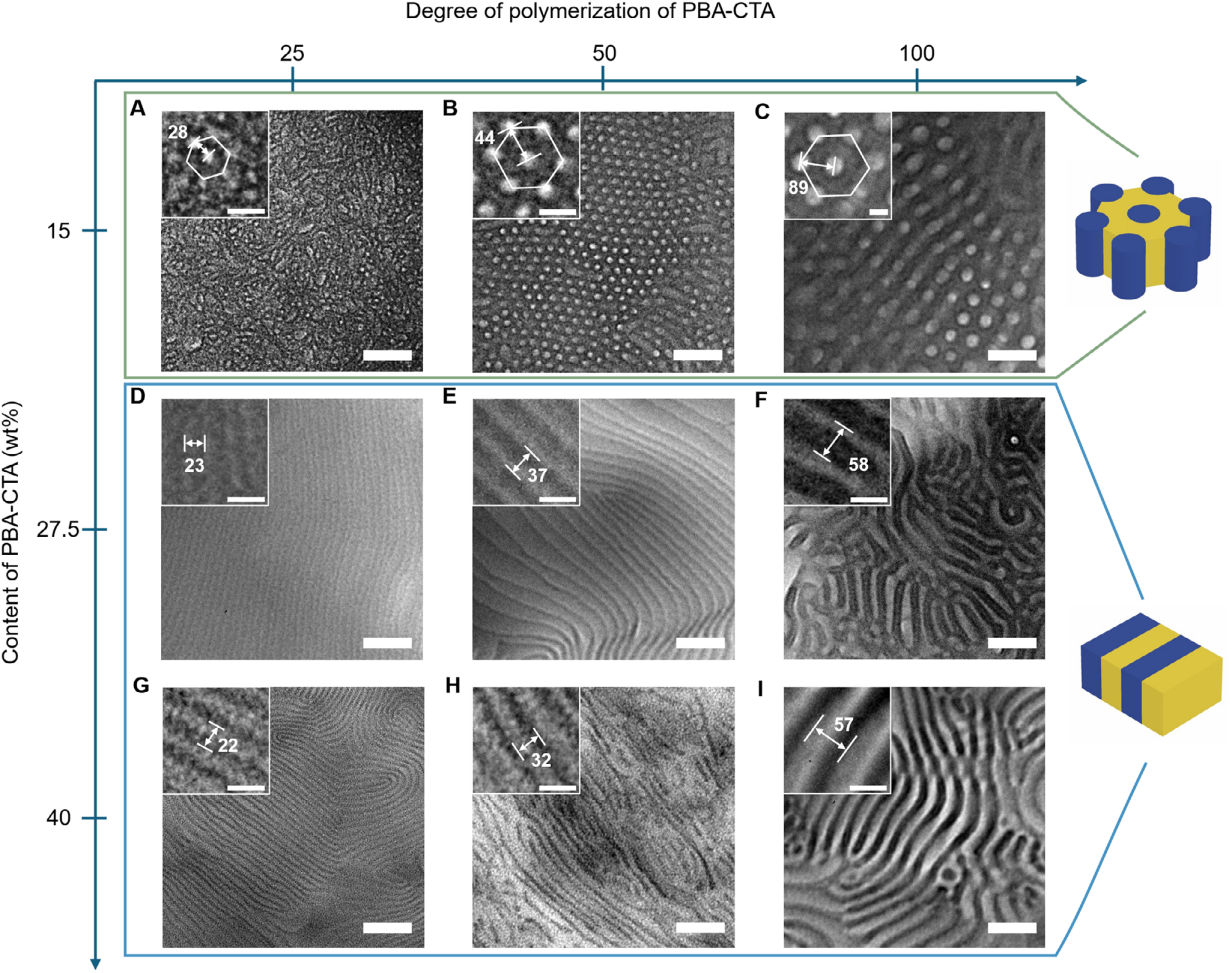
Transmission electron microscopy (TEM) micrographs of 3D printed PIANO materials after annealing (120°C 16 h). (A) 25/15.(B) 50/15.(C) 100/15.(D) 25/27.5.(E) 50/27.5.(F) 100/27.5.(G) 25/40.(H) 50/40.(I) 100/40.25/15 denotes the sample 3D printed from a resin containing 15.0 wt.% of PBA_25_‐CTA. Ultrathin sections were prepared by ultramicrotomy and selectively stained with osmium tetroxide (OsO_4_) prior to imaging. Scale bars are 200 nm for main images and 50 nm for insets. The brighter regions correspond to PBA‐rich domains, whereas the darker regions represent PAA‐rich domains. The plane‐to‐plane (*d*_p–p_) or cylinder center‐to‐center (*d*_c–c_) distances are indicated. Larger field‐of‐view images were provided in Figures S14–S22.

Representative micrographs highlighting the ordered nanostructures are displayed in Figure 4, with larger field‐of‐view images provided in Figures S14–S22. Unstained TEM images of the 100/15 formulation, shown in Figure S23, demonstrate that the ordered nanostructures are inherently present in the material. The application of OsO_4_ staining is found to primarily enhance image contrast, thereby facilitating clearer visualisation, rather than inducing or altering the underlying morphology. Insets provide high‐magnification views of specific morphological features. For formulations containing 15.0 wt.% macroCTA (Figure 4A–C), the nanostructures are predominantly cylindrical, displaying hexagonal or lamellar‐like patterns depending on the grain orientation. This is consistent with the HEX morphology predicted by the PAA volume fraction (*f*_PAA_ = 0.79) [32]. The center‐to‐center distance between adjacent cylinders (*d*_c–c_) increases systematically with macroCTA length, from 28 nm for 25/15 to 44 nm for 50/15 and to 89 nm for 100/15. To facilitate comparison with SAXS‐derived domain spacing (*d*_SAXS_), *d*_c–c_ values were converted into the cylinder‐to‐plane spacings (*d*_c–p_) using geometrical relationships (Figure S12) and summarized in Table 1.

As the macroCTA loading increases to 27.5 and 40.0 wt.% (Figure 4D–I), the morphology transitions from cylinders to lamellae, following the compositional shift toward a more symmetric block fraction. For the 25/27.5 and 25/40 formulations, TEM reveals long‐range ordered lamellar domains with comparable spacings of 23 and 22 nm, respectively. However, as the macroCTA DP increased from 25 to 100, the extent of long‐range order decreased and the grain orientation became more complex in accord with SAXS data. This reflects the inherent competition between thermodynamically driven ordering and the polymerization kinetics.

The *d*_TEM_ values marked in the TEM images and summarized in Table 1 represent average spacings extracted from regions displaying clearer ordering, providing a representative measure of the intrinsic lamellar structure. Notably, a gradual increase in lamellar spacing is observed with increasing macroCTA length, for example, at a fixed 27.5 wt.% macroCTA loading, *d*_TEM_ increased from 23 nm of 25/27.5 to 37 nm of 50/27.5 and to 58 nm of 100/27.5, showing excellent agreement with the corresponding SAXS data (Figure 3D–F).

Combined SAXS and TEM analyses confirm that the PIANO strategy enables the fabrication of highly ordered nanostructures within 3D‐printed materials [49], while offering systematic control over both morphology and domain spacing through formulation design. Specifically, transitions between lamellar (LAM) and hexagonally packed cylindrical (HEX) phases are observed as a function of the PAA‐to‐PBA volume fraction, demonstrating compositional control over nanoscale ordering. Together, these results establish an initial morphological map for these 3D‐printable resins that directly links formulation parameters to ordered nanostructures, providing a principled framework for the rational design of hierarchically ordered materials.

### 2.4 Programmable Mechanical Properties and Fabrication of Complex 3D Printed Objects

**FIGURE 5:**
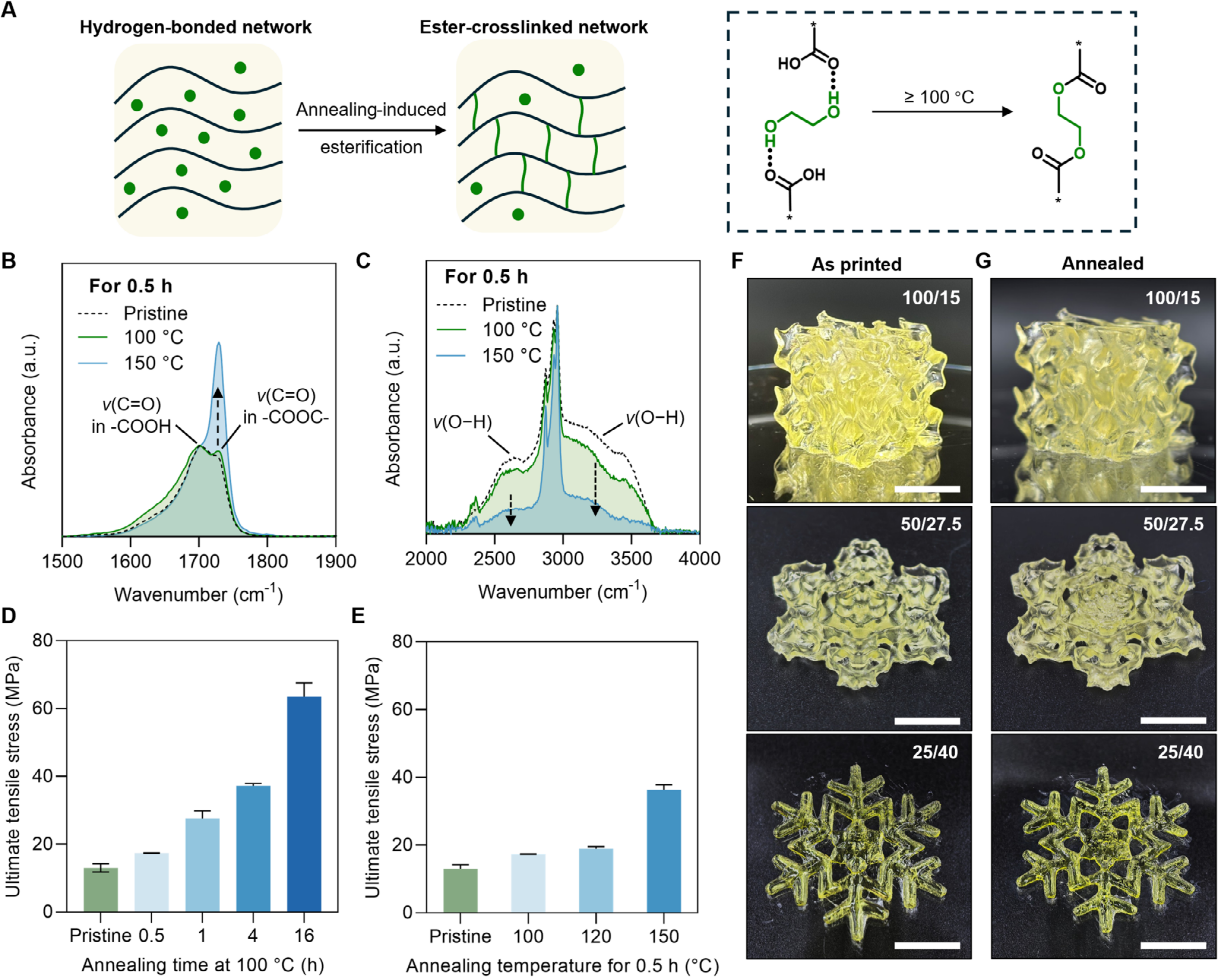
Programmable mechanical properties and fabrication of complex 3D printed objects. (A) Mechanisms of strength enhancement via esterification crosslinking during thermal annealing. FTIR spectra comparison of 3D printed PIANO samples 100/15 before and after thermal annealing at 100°C or 150°C for 0.5 h: (B) 1500–1900 cm^−1^ (normalized at 1702 cm^−1^) and (C) 2000–4000 cm^−1^ (normalized at 2959 cm^−1^). The increase in ultimate tensile stress with (D) longer annealing at 100°C and (E) higher temperature for 0.5 h. 3D printed complex objects using resin 100/15, 50/27.5, and 25/40, respectively: (F) as printed; (G) annealed. The scale bars are 10 mm.

Beyond their tunable ordered nanostructures, 3D‐printed PIANO materials exhibit programmable mechanical properties through post‐printing thermal treatments. The mechanism for this reinforcement is illustrated in Figure 5A: at elevated temperatures (≥ 100°C), the hydroxyl groups of EG react with the carboxylic acid groups of the PAA block, via in situ esterification, transforming the material into a robust, and covalently cross‐linked network. To investigate the effect of the temperature on the esterification reaction, we employed FTIR spectroscopy on 3D printed materials after annealing at 100°C or 150°C (Figure S24). As shown in Figure 5B, the absorption peak at 1702 cm^−1^ corresponds to the carbonyl stretching of carboxylic acid groups of PAA blocks, while the peak at 1729 cm^−1^ is attributed to the newly formed ester carbonyl groups [50]. We quantified the extent of esterification using the intensity ratio of the ester‐to‐acid carbonyl peaks (*I*_1729_/*I*_1702_; Table S6). After 0.5 h at 100°C, the ratio remained nearly identical to the pristine sample (0.87 vs. 0.86), indicating minimal reaction under mild conditions. However, increasing the temperature to 150°C led to an evident increase in this ratio. Further evidence of this chemical transformation is found in the 2000–4000 cm^−1^ region (Figure 5C). The pristine sample displays a broad O─H stretching band, characteristic of the dynamic hydrogen‐bonding network between EG and PAA. While this band decreased slightly after 100°C annealing, it became significantly narrower at 150°C, confirming the consumption of hydroxyl groups through covalent bond formation. Similarly, when the temperature was held constant at 100°C, the degree of covalent cross‐linking increased progressively with extended annealing time, as evidenced by the gradual increase in the *I*_1729_/*I*_1702_ ratio (Figure S25). This tunable cross‐linking density was further validated through swelling experiments in *N,N*‐dimethylformamide (DMF). As shown in Figure S26, pristine samples underwent complete dissolution, confirming the absence of permanent cross‐links. In contrast, annealed samples exhibited stable gel formation with a solvent resistance that increased progressively with annealing duration.

This esterification reaction provides an effective mechanism to tune the mechanical properties of 3D‐printed PIANO materials. To quantify this effect, we performed tensile testing on 3D‐printed dog‐bone specimens using the 100/15 formulation (Figure S27 and Table S7). As shown in Figure 5D, the ultimate tensile strength increased steadily from ∼13.0 MPa in the as‐printed state to ∼17.4 MPa after 0.5 h and reached ∼63.5 MPa, representing a 5‐fold increase, after 16 h of annealing at 100°C. Higher temperatures markedly accelerated these reinforcement kinetics; for example, annealing at 120°C increased the strength to ∼18.9 MPa, while annealing at 150°C resulted in a larger enhancement, reaching ∼35.5 MPa (Figure 5E). The esterification reaction and the resulting mechanical stiffening were also monitored in situ using dynamic mechanical analysis (DMA) under isothermal conditions. This non‐standard DMA protocol was intentionally designed to capture the time‐dependent evolution of mechanical properties during post‐printing crosslinking. During isothermal annealing, 100/15 samples exhibited a progressive increase in storage modulus, attributed to the continual formation of ester crosslinks at elevated temperatures. While reinforcement was modest at 100 and 120°C, it was highly pronounced at 150°C, where the modulus increased by 5.8‐fold, reflecting the rapid cross‐linking and network densification at elevated temperatures (Figure S28 and Table S8).

Finally, to demonstrate that the thermal annealing process preserved the macroscopic fidelity of the printed parts, we fabricated complex 3D geometries using three distinct formulations: 100/15, 50/27.5, and 25/40 (Figure 5F). These objects were subsequently annealed at 120°C for 16 h (Figure 5G). All printed objects maintained their designed macroscopic features and structural integrity after annealing. While the 25/40 formulation successfully formed ordered nanostructures, its lower initial mechanical strength slightly limited its capacity to support the most intricate three‐dimensional overhanging architectures during the printing process compared to the 100/15 system. Resolution testing (Figure S29 and Table S9) further revealed a minimum printable feature size of ∼200 µm, with no appreciable shrinkage upon thermal treatment. This dimensional stability is critical for the practical application of these materials in precision engineering. Collectively, these results demonstrate that the PIANO strategy enables robust multiscale structural control by seamlessly integrating well‐defined nanoscale ordering with the ability to print complex macroscopic geometries. Furthermore, the post‐printing esterification cross‐linking provides substantial mechanical reinforcement, effectively compensating for the initial strength reduction caused by the inclusion of the mobility mediator (EG). Together, these capabilities establish a solid foundation for the deployment of 3D‐printed materials with programmable ordered nanostructures.

To better understand the specific role of EG in mediating phase separation and mechanical properties, a non‐ethylene glycol control system was investigated using 1,2‐dimethoxyethane (DME) as an alternative mobility mediator (Table S10). SAXS measurements showed a primary scattering peak accompanied by a weak higher‐order reflection (Figure S30A), confirming that ordered nanostructures can form in the absence of EG. This demonstrates that the PIANO strategy is not limited to EG and that other small molecules with mobility‐enhancing capability can also facilitate ordered phase separation. However, the effectiveness of different mobility mediators depends not only on their molar ratio relative to the monomer, but also on specific molecular interactions with the polymer chains. EG, which contains two hydroxyl groups, can more effectively mediate hydrogen‐bonding interactions within PAA‐rich domains, thereby promoting segmental mobility during photopolymerization and leading to more pronounced structural ordering. Mechanical testing further highlights the distinct role of EG. Compared with the EG system, the DME sample showed slightly higher tensile strength (21.1 MPa), but significantly reduced elongation at break (21.8%) (Figure S30B and Table S11). In contrast, the more effective hydrogen bonding between EG and PAA blocks contributes to improved energy dissipation and enhanced ductility. Moreover, annealing resulted in only a modest strength increase in the DME system (24.4 MPa) vs. the pronounced reinforcement in the EG system (63.5 MPa), which is attributed to esterification‐induced crosslinking. Taken together, these results demonstrate that while the PIANO strategy is not restricted to EG, EG provides a unique combination of hydrogen‐bond‐mediated plasticization during phase separation and latent chemical locking upon annealing in this PIANO system.

## 3 Conclusion

This work establishes PIANO as an effective strategy to address the long‐standing kinetic mismatch between block‐copolymer ordering and the rapid curing rates inherent to vat photopolymerization‐based 3D printing. By leveraging ethylene glycol as a representative mobility mediator, sufficient segmental mobility is maintained during chain growth, enabling the formation of long‐range ordered nanostructures within bulk printed architectures directly under realistic 3D printing conditions, and thereby largely bypassing the conventional reliance on prolonged thermal annealing or solvent‐assisted processing. Importantly, PIANO allows nanoscale ordering to occur concomitantly with chain growth by decoupling polymerization from the crosslinking steps, achieving self‐assembly within seconds under mild conditions. This principle offers a potential versatile framework for any photopolymerizable system where phase separation competes with kinetic arrest.

By integrating ordered nanoscale self‐assembly into additive manufacturing workflows, PIANO transforms vat photopolymerization into a truly multiscale manufacturing paradigm, where nanoscale morphology and domain spacing are programmable via resin formulation. Beyond offering a practical route to architected materials with integrated nano‐ and macro‐scale features, PIANO establishes an applicable approach for directing hierarchical organization under kinetically constrained manufacturing conditions, opening new opportunities for creating next‐generation functional systems where hierarchical structure dictates ultimate performance.

## 4 Experimental Section

Experimental details are available in the Supporting Information.

## Conflicts of Interest

The authors declare no conflicts of interest.

## Supporting information




**Supporting File**: adma73395‐sup‐0001‐SuppMat.docx.

## Data Availability

The data that support the findings of this study are available from the corresponding author upon reasonable request.
