# Supplementary material for: Additive Manufacturing of Ordered Polymer Nanostructures
Source: Adv Mater. 2026 May 23;38(47):e73395. doi: 10.1002/adma.73395 (PMC13496074; doi:10.1002/adma.73395)
Supplement: Supplementary file 1 [file ADMA-38-e73395-s001.docx]

**Supporting Information**

**Additive Manufacturing of Ordered Polymer Nanostructures**

Di Wu^1^, Kun Zhou^1^, Kenny Lee^1^, Yuan Xiu^1^, Carol Hiu Wai Yan^2^, Khoon S. Lim^3^, and Cyrille Boyer^1*^

^1^School of Chemical Engineering, University of New South Wales, Sydney, NSW 2052, Australia.

^2^Electron Microscope Unit, Mark Wainwright Analytical Centre, University of New South Wales, Sydney, NSW 2052, Australia.

^3^School of Medical Sciences, University of Sydney, Sydney, Australia

Email: cboyer@unsw.edu.au

**Materials and Methods**

**Materials**

Acetonitrile (MeCN, RCI Labscan Limited, HPLC) and *N,N*-dimethylformamide (DMF, RCI Labscan Limited, RCI Premium) were used as received. 2-(*n*-butylthiocarbonothioylthio) propanoic acid (BTPA, Boron Molecular, >95%), diphenyl (2,4,6-trimethylbenzoyl) phosphine oxide (TPO, Sigma-Aldrich, >97%), 2,2’-azobis(2-methylpropionitrile) solution (AIBN solution, Sigma-Aldrich, 0.2 M in toluene), acrylic acid (AA, anhydrous, Sigma-Aldrich, ≥99%), ethylene glycol (EG, Sigma-Aldrich, ≥99%), 1,2-dimethoxyethane (DME, Sigma-Aldrich, ≥99%), and ethylene glycol diacrylate (EGDA, Sigma-Aldrich, ≥99%) were used as received. *n*-Butyl acrylate (BA, Sigma-Aldrich, ≥99%) was passed through a basic aluminium oxide column to remove inhibitor prior to use.

**Methods**

Synthesis of various macroCTAs (PBA*_n_*-CTAs)

The macroCTAs were synthesized by Reversible Addition-Fragmentation Chain Transfer (RAFT) polymerization of *n*-butyl acrylate in acetonitrile. Take PBA_100_-CTAs for example, *n*-butyl acrylate (40 g, 0.312 mol), BTPA RAFT agent (0.744 g, 3.12 × 10^-3^ mol) and AIBN (0.2 M solution in toluene, 2.34 mL, 4.68 × 10^-4^ mol) were dissolved in acetonitrile (80 mL). The mixture was deoxygenated by purging with nitrogen for 60 min, and then polymerized for 16 h at 60 °C. The reaction was stopped by exposure to air. The polymer solution was concentrated by rotary evaporation. Using the same protocol, PBA_50_-CTA and PBA_25_-CTA were synthesized.

3D printing setup

A typical procedure for fabricating 3D printed objects is as follows: A 3D object was designed using Tinkercad 3D modelling software and the object was exported as an .stl file format. The .stl file was opened using Photon Workshop, where the Z lift speed was set to 0.5 mm s^-1^ and Z retract speed was set to 1 mm s^-1^, while the Z lift distance was set to 5 mm. Printing parameters, such as layer thickness and exposure time, were defined in Photon workshop, sliced, and exported as .pws files for 3D printing. The .pws file copied to a flash drive for use with a masked LCD 3D printer (Anycubic Photon Mono SE) with a violet light LED array (*λ*_max_ = 405 nm, *I*_0_ = 2 mW cm^-2^). All samples were 3D printed using a layer thickness of 100 μm. The cure time per layer was 60 s for all the 3D printed samples, except for the 25/40 formulation, which required an extended cure time of 90 s. After 3D printing was completed, the printed objects were separated from a build plate, washed with ethanol and post-cured under violet light (*λ*_max_ = 405 nm, *I*_0_ = 10 mW cm^-2^) for 30 min to ensure complete monomer conversion.

Nuclear magnetic resonance (NMR)

All NMR spectra were recorded on Bruker Avance III 400 MHz spectrometer using an external lock (CDCl_3_).

Size exclusion chromatography (SEC)

Analysis of the molecular weight distributions of the polymers were determined using a Shimadzu modular system composed of an SIL-20A auto-injector, a Polymer Laboratories 5.0 μm bead-size guard column (50 × 7.5 mm^2^) followed by three linear PL (Styragel) columns (10^5^, 10^4^ and 10^3^), an RID-10A differential refractive-index (RI) detector, and a UV detector. The eluent was DMAc (containing 0.03% w/v LiBr and 0.05% w/v 2,6-dibutyl-4-methylphenol (BHT)) at 50 °C, run at a flow rate of 1.0 mL/min. The SEC was calibrated using narrow poly(methyl methacrylate) (PMMA) standards with molecular weights of 200 – 10^6^ g/mol.

Attenuated total reflectance-Fourier transform infrared (ATR-FTIR) spectroscopy

ATR-FTIR spectroscopy was performed using a Bruker Alpha FTIR spectrometer equipped with room temperature DTGS detectors.

To monitor photopolymerization kinetics, 15 μL of prepared resin was pipetted onto the ATR crystal plate after background scanning. An absorption spectrum was then obtained by scanning the droplet from 400-4000 cm^-1^. After an initial reading, the droplet was irradiated with a Thorlabs mounted LED with a collimation adapter (*λ*_max_ = 405 nm, *I*_0_ = 2 mW cm^-2^) for various times (0, 15, 30, 45, 60, 75, and 90 s) and subsequently the IR absorption spectra were obtained to determine the integral of the vinylic peak at time *t_x_*. Vinyl bond conversions were calculated from the disappearance of the C=C stretching peak (1585-1650 cm^-1^) normalized to the C=O stretching peak (1650-1760 cm^−1^) as an internal standard using **Supplementary Equation S1**:

$Conversion (\%) = 100 \times( 1- {\frac{{int}_{x}}{{std}_{x}}}/{\frac{{int}_{0}}{{std}_{0}}})$	**(S1)**

Where *int_x_* is the integral of the 1585-1650 cm^-1^ peak at time *t_x_* of irradiation, *std_x_* is the integral of the 1650-1760 cm^-1^ peak at time *t_x_* of irradiation, *int_0_* is the initial integral of the 1585-1650 cm^-1^ peak before irradiation, and *std_0_* is the initial integral of the 1650-1760 cm^-1^ peak before irradiation.

To monitor sample changes after annealing treatment, the 3D printed PIANO samples (6 × 6 × 2 mm) were fixed onto the ATR crystal plate after annealing. An absorption spectrum was then obtained by scanning the samples from 400-4000 cm^-1^.

Photorheology

Photorheological measurements were conducted using a rheometer (Anton Paar MCR 301) equipped with a stainless-steel measuring plate (25 mm diameter), a glass plate with Peltier temperature control (P-PTD 200/GL), and an integrated UV light source (OmniCure Series 1500).

For sample preparation, 0.5 mL of precursor solution was aliquoted onto the glass plate, after which the measuring plate was lowered to a gap of 0.2 mm. Excess solution was removed once the desired gap was achieved.

Each curing experiment consisted of two intervals. In the first interval, no UV irradiation was applied to allow the system to reach a stable baseline. In the second interval, UV light was applied to initiate curing. During UV exposure, oscillatory measurements were performed at a shear strain of 0.1% and an angular frequency of 6.28 rad s⁻¹. The irradiation wavelength was filtered to 405 nm with an intensity of 10.54 mW cm⁻².

Small-angle X-ray scattering (SAXS)

SAXS experiments were performed on an Anton Paar SAXSPoint 2.0 system with a Cu K_α_ (*λ* = 0.154 nm) microfocus X-ray source (50 kV/1 mA) and Dectris Eiger 1M detector. Data was collected at room temperature, under vacuum with a sample-to-detector distance of 0.575 m. Samples were 3D printed at the thickness of 3×100 μm layers. Data was reduced to 1D by radial averaging the 2D detector after converting pixel positions to *q* = (4π/*λ*)sin*θ*, where 2*θ* is the scattering angle). The domain spacing was calculated using **Supplementary Equation S2**:

$d_{SAXS}= \frac{2\pi}{q^{*}}$	**(S2)**

As shown in **Figure S5**, for nanostructures with hexagonal packed cylinders (HEX), the calculated *d*_SAXS_ by **Equation S2** corresponds to the distance between adjacent lattice planes, or from a cylinder center to the nearest lattice plane (*d*_c-p_), instead of center-to-center distance between adjacent cylinders (*d*_c-c_); for nanostructures with lamellae (LAM), the calculated *d*_SAXS_ by **Equation S2** gives the lamellar period, center-to-center distance between domains of the same phase, or the interface-to-interface spacing across both phases (*d*_i-i_).

Fourier transform near-infrared (FTNIR) spectroscopy

FTNIR spectroscopy was performed using a Bruker Vertex 70 Fourier transform spectrometer. FTNIR spectroscopy was used to determine vinyl bond conversion of solid samples by comparing the integral of the C-H vinylic stretching overtone at 6120-6220 cm^-1^ between solid printed samples and a sample of uncured resin. Uncured resin (3 mL) was placed in a 1×1 cm quartz cuvette, and an absorption spectrum was obtained by scanning from 4000-8000 cm^-1^. A solid 3D printed sample was measured using a digital caliper, placed in the cuvette and another absorption spectra was measured. The final conversion was calculated using **Supplementary Equation** **S3:**

$Conversion (\%) = 100 \times( 1- {\frac{{int}_{f}}{t_{sample}}}/{\frac{{int}_{0}}{t_{cuvette}}})$	**(S3)**

Where *int_f_* is the integral of the peak from 6120-6220 cm^-1^ for the 3D printed sample, *int_0_* is the integral of the peak from 6120-6220 cm^-1^ for the unpolymerized resin, *t_sample_* is the thickness of the sample, and *t_cuvette_* is the path length of the cuvette. Integrals were calculated using OPUS software 7.5.

Rheological characterization

Rheological characterization of the resins was performed on a TA instrument AR1500ex Rheometer equipped with a 40 mm diameter parallel steel plate geometry at a 500 μm gap at 25 °C. Approximately 2 ml of resin was used for each measurement. Three sets of tests measurements were performed. Steady-shear flow curves were obtained by measuring viscosity between shear range of 100 and 10 1/s.

Transmission electron microscopy (TEM)

Cubic TEM samples with an edge length of 2 mm were 3D printed. After post-curing, the cubic samples were annealed at 120 °C for 16 h. Subsequently, cubic samples were embedded in Lowicryl HM20 resin, cured at -15 °C, then sectioned at ambient temperature to 70 nm thickness using Leica EM UC7 ultramicrotome with a DiATOME diamond knife. Sections were transferred onto carbon formvar copper grids. Prior to imaging, staining was performed by exposure of copper grids to the vapor of 2% osmium tetroxide solution for 15minutes. TEM grids were observed in a Thermo Fisher Scientific Talos L120C transmission electron microscope, operated at 120kV.

As shown in **Figure S5**, for nanostructures with hexagonal packed cylinders (HEX), the *d*_TEM_ was obtained by converting the measured center-to-center distance between adjacent cylinders (*d*_c-c_) into the distance from a cylinder center to the nearest lattice plane (*d*_c-p_), using geometrical relationships (**Figure S5**); for nanostructures with lamellae (LAM), the *d*_TEM_ was measured by the lamellar period, center-to-center distance between domains of the same phase, or the interface-to-interface spacing across both phases (*d*_i-i_).

Tensile testing

Tensile properties were measured using a Mark–10 ESM303 with a 2500 N force gauge model M5–200 at a deformation rate of 2.1 mm min^-1^. Dog-bone-shaped samples were 3D printed with a length of 35 mm, width of 2 mm and gauge length of 12 mm. All tensile results were performed in at least triplicate. The tensile stress was calculated from the applied force divided by the initial cross-sectional area of the gage section. The strain was determined as the change in gage length relative to the original specimen gage length, expressed as a percentage. Young’s modulus was determined by the slope of the linear elastic region. Elongation at break was determined from the strain corresponding to the fracture point in the stress-strain curve. Toughness was determined by calculating the area under a stress-strain curve using the trapezoidal rule.

Dynamic mechanical analysis (DMA)

DMA was performed using a TA instruments Q800 dynamic mechanical analyzer which was equipped with a TA instruments liquid nitrogen gas cooling accessory (GCA) for temperature control. The 3D printed samples (35 × 8 × 2 mm) were measured using digital calipers, placed into the single cantilever clamp (17.5 mm), and fixed. The samples were heated to the target temperature (100, 120, and 150 °C, respectively), allowed to equilibrate for 1 min, and subsequently held isothermally for 2 h with constant frequency of 1 Hz and displacement of 20 μm. The isothermal mode was conducted to monitor the time-independent increase in storage modulus at a constant temperature for 2 h.

Swelling testing

The 3D printed samples (6 mm × 6 mm × 1 mm) were immersed in DMF in a small vial for over 2 h to reach equilibrium.

**Supplementary figures and tables**

**Figure S1.** Synthesis of PBA*_n_*-CTAs (macroCTAs) via thermal Reversible Addition-Fragmentation Chain Transfer (RAFT) polymerization of *n*-butyl acrylate using BTPA as the RAFT agent, yielding well-defined macroCTAs for subsequent PIANO and PIMS processes.

**Figure S2.** Molecular weight distributions of PBA*_n_*-CTAs obtained by SEC using a refractive index (RI) detector. The number-average molecular weights (*M*_n_) and dispersity (*Đ*) for PBA_25_-CTA, PBA_50_-CTA, and PBA_100_-CTA are 3.8, 5.6, and 11.0 kDa, respectively, as summarized in **Table S1**.

**Figure S3.** ^1^H NMR spectra (400 MHz, CDCl_3_, 298 K). (A) ^1^H NMR spectra of crude PBA*_n_*-CTAs macroCTAs; (B) Example of calculation of monomer conversion using ^1^H NMR analysis. ^1^H NMR spectrum of crude PBA_100_-CTA. The BA conversion is 97% in this specific example. The BA monomer conversion was determined by ^1^H NMR using the residual vinyl signals (5.5–6.5 ppm, *I*_1_,*I*_2_ and *I*_3_, 3H) and the –OCH_2_– signal at 4.03 ppm (2H, *I*_a_), according to $X_{\mathrm{BA}} (\%)=100-{[(I}_{\text{1}}+I_{\text{1}}+I_{\text{1}})/3]/(I_{a}/2)\times100 \%$.

**Table S1.** Characterization of prepared PBA*_n_*-CTAs (macroCTAs).

| No. | MacroCTA | Conversion  (%)^a^ | *M*_n, NMR_  (kg mol^-1^)^b^ | *M*_n, SEC_  (kg mol^-1^) | *Đ* |
| --- | --- | --- | --- | --- | --- |
| 1 | PBA_25_-CTA | 96 | 3.3 | 3.8 | 1.1 |
| 2 | PBA_50_-CTA | 97 | 6.5 | 5.6 | 1.1 |
| 3 | PBA_100_-CTA | 97 | 12.7 | 11.0 | 1.1 |

^a^Monomer conversion was calculated by ^1^H NMR spectroscopy (**Figure S3B**, SI).

^b^*M*_n, NMR_ = Target DP × conv. (BA) × MW(BA) + MW(BTPA).

**Table S2.** Formulations of PIANO resins at a fixed [AA]/[EG] molar ratio of 6.4.

| No. | MacroCTAs | Resins | Resin component (ꞷ, wt%) | | | | *f*_PAA_^a^ |
| --- | --- | --- | --- | --- | --- | --- | --- |
|  |  |  | MacroCTA | AA | EG | TPO |  |
| 1 | PBA_25_-CTA | 25/15 | 15.0 | 74.0 | 10.0 | 1.0 | 0.79 |
| 2 |  | 25/27.5 | 27.5 | 63.0 | 8.5 | 1.0 | 0.64 |
| 3 |  | 25/40 | 40.0 | 52.0 | 7.0 | 1.0 | 0.50 |
| 4 | PBA_50_-CTA | 50/15 | 15.0 | 74.0 | 10.0 | 1.0 | 0.79 |
| 5 |  | 50/27.5 | 27.5 | 63.0 | 8.5 | 1.0 | 0.64 |
| 6 |  | 50/40 | 40.0 | 52.0 | 7.0 | 1.0 | 0.50 |
| 7 | PBA_100_-CTA | 100/15 | 15.0 | 74.0 | 10.0 | 1.0 | 0.79 |
| 8 |  | 100/27.5 | 27.5 | 63.0 | 8.5 | 1.0 | 0.64 |
| 9 |  | 100/40 | 40.0 | 52.0 | 7.0 | 1.0 | 0.50 |
| 10 | PBA_25_-CTA | 25/10 | 10.0 | 78.4 | 10.6 | 1.0 | 0.86 |
| 11 |  | 25/7.5 | 7.5 | 80.6 | 10.9 | 1.0 | 0.89 |

^a^Density values: 𝜌_PBA_ = 1.08 g cm^-3^; 𝜌_PAA_ = 1.41 g cm^-3^. The volume fraction *f*_PAA_ was calculated according to the following equations.

$V= \frac{\omega}{\rho}$	**(E3)**

$f_{PAA}= \frac{V_{PAA}}{V_{PAA}+V_{PBA}}$	**(E4)**

**Figure S4.** Polymerization kinetics study of PIANO resins measured by ATR-FTIR spectroscopy. Vinyl conversion versus time for different formulations.

Note:

**Note:** A 15 µL droplet was exposed to irradiation using a Thorlabs mounted LED equipped with a collimation adapter (*λ*_max_ = 405 nm, *I*_0_ = 2 mW cm^2^) for different durations: 0, 15, 30, 45, 60, 75, and 90 seconds. Measurements were taken immediately following each exposure period. Each data point reflects the average result from three independent experiments (n = 3), ensuring reproducibility and statistical reliability.

**Figure S5.** Photorheology of the PIANO 50/15 resin showing the evolution of storage modulus and loss modulus during photopolymerization. The rapid modulus increases following light exposure indicates a transition from liquid-like to solid-like behavior associated with polymerization-induced solidification. Each curing experiment consisted of two intervals. In the first interval, no UV irradiation was applied to allow the system to reach a stable baseline. In the second interval, UV light was applied to initiate curing. During UV exposure, oscillatory measurements were performed at a shear strain of 0.1% and an angular frequency of 6.28 rad s⁻¹. The irradiation wavelength was filtered to 405 nm with an intensity of 10.54 mW cm⁻².

**Figure S6.** *In situ* SAXS profiles of 100/15 PIANO resin during photopolymerization. With increasing irradiation time, the primary scattering peak gradually shifts to lower *q*, indicating an increase in the domain spacing. Concurrently, the evolution of higher-order reflections from a 1:2 to a 1:√3 ratio relative to the primary peak suggests a transition in microphase phase separation from lamellar to hexagonally packed cylindrical morphologies hexagonal.

**Table S3.** Formulations of PIMS resins.

| No. | MacroCTA | Resin | Resin composition (wt%) | | | |
| --- | --- | --- | --- | --- | --- | --- |
|  |  |  | MacroCTA | AA | EGDA | TPO |
| 1 | PBA_50_-CTA | 50/15 | 15.0 | 61.3 | 22.7 | 1.0 |
| 2 | PBA_25_-CTA | 25/40 | 40.0 | 43.0 | 16.0 | 1.0 |

^*^In the PIMS formulations, the molar ratio of [AA]/[EGDA] was fixed at 6.4, corresponding to the same [AA]/[EG] molar ratio used in the PIANO formulations.

**Figure S7.** FTNIR spectra of an unpolymerized 50/15 resin and a 3D printed 50/15 sample after post-curing. The near-complete disappearance of the vinylic C-H stretching overtone at ~6200 cm^-1^ suggests essentially complete conversion of vinyl monomers.

**Figure S8.** Representative simple 3D printed objects with formulations 50/15, 50/27.5, and 50/40. The scale bars are 10 mm.

**Table S4.** Resin viscosity of formulations 50/15, 50/27.5, and 50/40 measured at 25 °C and a shear rate of 50 s^-^¹.

| Resin | 50/15 | 50/27.5 | 50/40 |
| --- | --- | --- | --- |
| Viscosity (Pa s) | 0.0126 | 0.0254 | 0.0546 |

**Figure S9.** SAXS profiles of 3D printed PIANO samples using PBA_25_ macroCTA at 10 and 7.5 wt% content. The detailed formulations were listed in **Table S2**. The higher-order scattering with peak positions at q and √3q corresponds to hexagonally packed cylindrical domains.

**Table S5.** Formulation compositions corresponding to **Figure S8.**

| No. | Legend  in **Figure S8** | MacroCTA | Resin component (wt%) | | | |
| --- | --- | --- | --- | --- | --- | --- |
|  |  |  | MacroCTA | AA | solvent | TPO |
| 1 | 0 wt% | PBA_100_-CTA | 15.0 | 84.0 | 0.0 | 1.0 |
| 2 | 5 wt% |  | 15.0 | 79.0 | 5.0 | 1.0 |
| 3 | 10 wt% |  | 15.0 | 74.0 | 10.0 | 1.0 |
| 4 | 15 wt% |  | 15.0 | 69.0 | 15.0 | 1.0 |
| 5 | 100/40 | PBA_100_-CTA | 40.0 | 52.0 | 7.0 | 1.0 |
| 6 | Increased EG |  | 37.0 | 49.0 | 13.0 | 1.0 |
| 7 | Reduced TPO |  | 40.2 | 52.3 | 7.0 | 0.5 |

**Figure S10.** Formulation-dependent modulation of nanoscale ordering in the PIANO process. (A) SAXS profiles of 3D printed PIANO samples prepared from the modified 100/15 formulation with systematically varied ethylene glycol (EG) weight fractions (0, 5, 10 and 15 wt%). (B) SAXS profiles of 3D printed PIANO samples prepared from 100/40 and modified 100/40 formulations. Two modified formulations were investigated: (i) increased EG content and (ii) reduced TPO concentration. The detailed formulation compositions are listed in **Table S5**.

**Figure S11.** Power law scaling of domain spacings in 3D printed PIANO materials before annealing. Log-log plot of domain spacing (*d*_SAXS_) as a function of total degree of polymerization (*N*'_total_) across three macroCTA loadings (15.0, 27.5, and 40.0 wt%). *d*_SAXS_ determined from SAXS, and *N*'_total_ was calculated based on a common monomer reference volume (118 Å^3^). Power law regression lines are included to highlight the strong scaling relationship. The scaling exponents obtained were similar for all three loadings of macroCTA, giving a consistent exponent of δ ≈ 0.69 (R^2^ ≈ 0.998–0.999), indicating a robust and predictable dependence of nanoscale feature size on polymerization degree.

Note: In classical block copolymer theory, δ = 1/2 is expected in the weak-segregation limit (Gaussian chains), whereas δ approaches 2/3 in the strong-segregation limit (SSL) where chains are increasingly stretched. The experimentally observed δ ≈ 0.69 is therefore close to the strong-segregation scaling, suggesting pronounced chain stretching and well-developed segregation in our system. The slight deviation from the ideal SSL value may arise from the effective definition of *N* in the polymerization system and from nonequilibrium effects inherent to *in situ* fabrication, yet the near-identical exponents obtained across different loadings highlight the reliability of this scaling law as a practical design rule for predicting domain spacings in 3D-printed materials.

**Figure S12.** Schematic illustration of domain spacing *d* in ordered nanostructures and the relationships among them and with *q*^*^ of SAXS profiles. (A) *d*_c-c_ in HEX is the center-to-center distance between cylinders. (B) *d*_c-p_ in HEX corresponds to the distance between adjacent lattice planes (from a cylinder center to the nearest lattice plane), used as the domain spacing *d*_TEM_ in TEM. (C) *d*_i-i_ in LAM corresponds to the full repeat period, center-to-center distance between domains of the same phase, or the interface-to-interface spacing across both phases.

**Figure S13.** SAXS profiles of 3D printed PIANO 100/15 samples before and after various annealing treatments. Note: The peak positions and overall peak shapes remain essentially unchanged, confirming that the ordered nanoscale morphology is preserved during the annealing treatment.

**Figure S14.** TEM micrographs of 3D printed PIANO 25/15 sample after annealing. Representative micrographs obtained from ultramicrotomed cross-sections show the internal morphology of two different areas. The red dashed line outlines a representative region of long-range order, reported in Figure 4 (Main manuscript).

**Figure S15.** TEM micrographs of 3D printed PIANO 50/15 sample after annealing. Representative micrographs obtained from ultramicrotomed cross-sections show the internal morphology of two different areas. The red dashed line outlines a representative region of long-range order, reported in Figure 4 (Main manuscript).

**Figure S16.** TEM micrographs of 3D printed PIANO 100/15 sample after annealing. Representative micrographs obtained from ultramicrotomed cross-sections show the internal morphology of two different areas. The red dashed line outlines a representative region of long-range order, reported in Figure 4 (Main manuscript).

**Figure S17.** TEM micrographs of 3D printed PIANO 25/27.5 sample after annealing. Representative micrographs obtained from ultramicrotomed cross-sections show the internal morphology of two different areas. The red dashed line outlines a representative region of long-range order, reported in Figure 4 (Main manuscript).

**Figure S18.** TEM micrographs of 3D printed PIANO 50/27.5 sample after annealing. Representative micrographs obtained from ultramicrotomed cross-sections show the internal morphology of two different areas. The red dashed line outlines a representative region of long-range order, reported in Figure 4 (Main manuscript).

**Figure S19.** TEM micrographs of 3D printed PIANO 100/27.5 sample after annealing. Representative micrographs obtained from ultramicrotomed cross-sections show the internal morphology of two different areas. The red dashed line outlines a representative region of long-range order, reported in Figure 4 (Main manuscript).

**Figure S20.** TEM micrographs of 3D printed PIANO 25/40 sample after annealing. Representative micrographs obtained from ultramicrotomed cross-sections show the internal morphology of two different areas. The red dashed line outlines a representative region of long-range order, reported in Figure 4 (Main manuscript).

**Figure S21.** TEM micrographs of 3D printed PIANO 50/40 sample after annealing. Representative micrographs obtained from ultramicrotomed cross-sections show the internal morphology of two different areas. The red dashed line outlines a representative region of long-range order, reported in Figure 4 (Main manuscript).

**Figure S22.** TEM micrographs of 3D printed PIANO 100/40 sample after annealing. Representative micrographs obtained from ultramicrotomed cross-sections show the internal morphology of two different areas. The red dashed line outlines a representative region of long-range order, reported in Figure 4 (Main manuscript).

**Figure S23.** TEM micrographs of 3D printed PIANO 100/15 sample after annealing without staining. Representative micrographs obtained from ultramicrotomed cross-sections show the internal morphology of two different areas, confirming that the staining method does not affect the nanostructuration of our samples.

**Figure S24.** FTIR spectra for 3D printed PIANO 100/15 samples subjected to various annealing treatments. (A) full spectra ranging from 400 to 4000 cm^-1^ and (B) enlarged region (1500−2000 cm^-1^). The reduction in the carboxylic acid band intensity at 1702 cm^-1^ indicates the progression of the esterification process during thermal treatment.

**Table S6.** **Summary of FTIR peak intensities and esterification ratios.** The table lists the absorption intensities at 1702 cm⁻¹ (carbonyl of carboxylic acid) and 1729 cm⁻¹ (carbonyl of ester) for 3D-printed PIANO 100/15 samples following various annealing protocols. The degree of esterification is estimated by the intensity ratio I_1729_/I_1702_ to minimize sample-to-sample variation.

| No. | Annealing conditions | | Peak intensity | | Intensity ratio |
| --- | --- | --- | --- | --- | --- |
|  | Temperature (°C) | Duration (h) | 1702 cm^-1^ | 1729 cm^-1^ |  |
| 1 | Pristine | | 0.397 | 0.342 | 0.86 |
| 2 | 100 | 0.5 | 0.404 | 0.351 | 0.87 |
| 3 | 150 | 0.5 | 0.236 | 0.504 | 2.13 |

**Note:** The peak intensity refers to the peak height.

**Figure S25.** FTIR spectra ranging from 1500 to 1900 cm^-1^ (normalized at 1702 cm^-1^) for 3D printed PIANO 100/15 samples after annealing at 100 °C for different reaction times. The increasing intensity ratio of the ester C=O band at 1729 cm^-1^ to the carboxylic acid band intensity at 1702 cm^-1^ indicates the progression of the esterification process during thermal treatment.

**Figure S26.** (A) Swelling behaviors in DMF for 3D printed PIANO 100/15 samples after annealing at 100 °C for different reaction times, showing a transition from an initially fully dissolved, homogeneous solution to a gel-like state with increasing annealing time. (B) Side view of the gel (annealing for 1 h) in a tilted position.

**Figure S27.** Representative tensile curves of 3D printed PIANO 100/15 samples before and after different annealing treatments.

**Table S7.** Summary of mechanical properties of 3D printed PIANO 100/15 samples before and after different annealing treatments.

| No. | Annealing conditions | | Ultimate tensile stress (MPa) | Elongation  (%) | Toughness  (MJ m^-3^) |
| --- | --- | --- | --- | --- | --- |
|  | Temperature (°C) | Duration (h) |  |  |  |
| 1 | Pristine | | 13.0 ± 1.2 | 353.0 ± 43.3 | 32.6 ± 2.9 |
| 2 | 100 | 0.5 | 17.4 ± 0.1 | 480.0 ± 68.6 | 48.3 ± 5.8 |
| 3 | 100 | 1 | 27.6 ± 2.2 | 75.0 ± 22.0 | 13.2 ± 3.7 |
| 5 | 100 | 4 | 37.2 ± 0.7 | 31.0 ± 1.4 | 5.8 ± 0.5 |
| 6 | 100 | 16 | 63.5 ± 4.0 | 38.4 ± 0.4 | 9.8 ± 0.3 |
| 7 | 120 | 0.5 | 18.9 ± 0.7 | 20.6 ± 2.5 | 1.7 ± 0.2 |
| 8 | 150 | 0.5 | 35.5 ± 0.6 | 34.5 ± 6.0 | 4.6 ± 1.0 |

**Figure S28.** Dynamic Mechanical Analysis (DMA) profiles obtained in the isothermal mode for the 3D printed PIANO 100/15 samples, recorded at 100, 120, and 150 °C for 2 h, showing the time-dependent evolution of storage modulus at the specified temperature.

**Table S8.** Summary of DMA results in isothermal mode for 3D printed PIANO 100/15 samples.

| Sample | Isothermal protocol | Initial storage modulus (MPa)^a^ | Final storage modulus (MPa)^b^ | Increase ratio^c^ |
| --- | --- | --- | --- | --- |
| 100/15 | At 100 °C for 2 h | 2.99 | 3.99 | 1.33 |
|  | At 120 °C for 2 h | 2.39 | 5.13 | 2.15 |
|  | At 150 °C for 2 h | 1.39 | 8.13 | 5.85 |

^a^ Measured at the beginning of the isothermal annealing process.

^b^ Measured after 2 h of isothermal annealing at the indicated temperature.

^c^ The ratio of the final storage modulus to the initial storage modulus.

**Figure S29.** **Resolution testing of 3D-printed PIANO materials.** Representative lattice structures printed using 100/15 and 50/27.5 resins in the (**A**) as-printed state and (**B**) after thermal annealing (120 °C for 16 h). Scale bars represent 1 mm. The diameters of selected pillars were measured using ImageJ; the resulting data, confirming minimal shrinkage and high geometric fidelity, are summarized in **Table S9**.

**Table S9.** Comparison of the selected pillar top-face side length before and after annealing.

| Sample | Pillar number | Side length (µm) | |
| --- | --- | --- | --- |
|  |  | As printed | Annealed |
| 100/15 | 1 | 889 | 852 |
|  | 2 | 436 | 411 |
|  | 3 | 161 | 145 |
| 50/27.5 | 1 | 842 | 828 |
|  | 2 | 474 | 441 |
|  | 3 | 210 | 176 |

**Table S10.** Formulations of PIANO system with 1,2-dimethoxyethane (DME) as mobility mediator.

| No. | MacroCTAs | [AA]/[DME] molar ratio | Resin component (wt%) | | | |
| --- | --- | --- | --- | --- | --- | --- |
|  |  |  | MacroCTA | AA | DME | TPO |
| 1 | PBA_100_-CTA | 6.4 | 15.0 | 70.3 | 13.7 | 1.0 |

**Figure S30.** PIANO system with DME as mobility mediator. (A) SAXS profiles of 100/15 samples with [AA]/[DME] molar ratio at 6.4. (B) Representative tensile profiles of 100/15 samples with [AA]/[DME] molar ratio at 6.4 before and after annealing treatment (75^o^C, 16 h).

Note: The temperature was fixed at 75 ^o^C due to the low boiling point of DME (85 ^o^C).

**Table S11.** Summary of mechanical properties of 3D printed PIANO 100/15 samples with DME as mobility mediator before and after annealing treatments.

| No. | Annealing conditions | | Ultimate tensile stress (MPa) | Elongation  (%) | Toughness  (MJ m^-3^) |
| --- | --- | --- | --- | --- | --- |
|  | Temperature (°C) | Duration (h) |  |  |  |
| 1 | Pristine | | 21.1 ± 2.2 | 21.8 ± 0.5 | 2.1 ± 0.2 |
| 2 | 75 | 16 | 24.4 ± 0.3 | 27.4 ± 0.7 | 2.6 ± 0.2 |
